# Impact of prepared vascular access on mortality and medical expenses in elderly and non-elderly Japanese patients with chronic kidney disease stage G5: a retrospective cohort study

**DOI:** 10.1007/s10157-025-02654-3

**Published:** 2025-03-18

**Authors:** Takayuki Nimura, Makoto Harada, Daiki Aomura, Kosuke Yamaka, Koji Hashimoto, Yuji Kamijo

**Affiliations:** Department of Nephrology, Shinshu University School of Medicine, 3-1-1, Asahi, Matsumoto, 390–8621 Japan

**Keywords:** Elderly patients, Hemodialysis, Hospitalization expenses, Mortality, Vascular access

## Abstract

**Background:**

Patients with chronic kidney disease (CKD) stage 5 (CKDG5) have greater dialysis requirements that increase the risk of cardiovascular disease and mortality. The elevated costs associated with CKDG5 are a serious concern. The impact of prepared vascular access (VA) through planned VA creation on mortality and medical expenses remains unclear in Japanese patients with CKDG5.

**Methods:**

We conducted a retrospective cohort study including 157 patients with CKD who started hemodialysis (HD) at Shinshu University Hospital from April 2016 to March 2021 and assessed the relationship between the presence of a prepared VA and mortality and hospitalization expenses in elderly and non-elderly patients with CKDG5.

**Results:**

The presence of a prepared VA was associated with lower mortality in non-elderly patients but not in elderly patients. Medical expenses, emergency HD, and hospitalization duration were significantly lower in patients with a prepared VA in both age groups. The contribution of a prepared VA to mortality and medical expenses remained consistent after adjusting for sex, performance status, comorbidities, and nutritional status.

**Conclusion:**

A prepared VA showed several benefits, including lower mortality rates and hospitalization costs; shorter hospital stays; and higher home discharge rates. Planned VA creation was significantly associated with lower hospitalization expenses, irrespective of age.

**Supplementary Information:**

The online version contains supplementary material available at 10.1007/s10157-025-02654-3.

## Introduction

Chronic kidney disease (CKD) is a major health concern worldwide. As CKD progresses, the risk of end-stage kidney disease (ESKD) increases, ultimately requiring renal replacement therapy and increasing the risk of cardiovascular disease and mortality [1–3]. In more than 60% of patients with CKD stage G5 (CKDG5) dialysis is initiated within approximately 2 years after diagnosis [4]. A seamless transition to renal replacement therapy is reportedly crucial for patients with CKDG5. In patients who select hemodialysis (HD) as renal replacement therapy, creating vascular access (VA) before HD initiation is beneficial for improving their life expectancy [5–9]. Planned VA creation may prevent using a temporary central venous catheter for HD and decrease catheter-related adverse events, potentially shortening the hospital stay. Patients with CKDG5 without a prepared VA may have a longer hospital stay due to catheter-related adverse events as well as VA creation after HD initiation, resulting in increased hospitalization expenses. In Japan, healthcare services are provided under a universal health insurance system wherein the majority of medical expenses are covered by public insurance, with patients typically paying 30% of the costs. This may differ from healthcare systems in other countries, where cost-sharing mechanisms and insurance structures vary widely.

A prepared VA, such as an arteriovenous fistula (AVF) or arteriovenous graft (AVG), can increase cardiac output, resulting in maladaptive cardiovascular remodeling and heart failure due to increased preload by the shunt flow. Moreover, VA dysfunction due to stenosis or obstruction may occur before HD induction, leading to limited usefulness or disuse of the VA. Furthermore, considering the growing population of elderly patients with CKD undergoing HD, a prepared VA may be underutilized during their lifespan. Therefore, the usefulness of planned VA creation remains unclear, particularly in elderly patients with CKDG5. Identifying the impact of a prepared VA in these patients at HD initiation will enable us to evaluate the effect of planned VA creation.

This study investigated the relationship between a prepared VA and mortality as the primary outcome and hospitalization expenses as the secondary outcome. We also evaluated the differences in these relationships between elderly and non-elderly patients with CKDG5 to contribute to personalized medicine. To investigate the prognostic factors in patients with CKDG5, we examined the specific clinical factors in this population, including emergent initiation of dialysis, clinical trajectory of kidney dysfunction such as acute kidney injury (AKI) in CKD, type of final VA, sex, nutritional status, comorbidity, and performance status (PS).

## Materials and methods

### Study design and patients

**Fig. 1:**
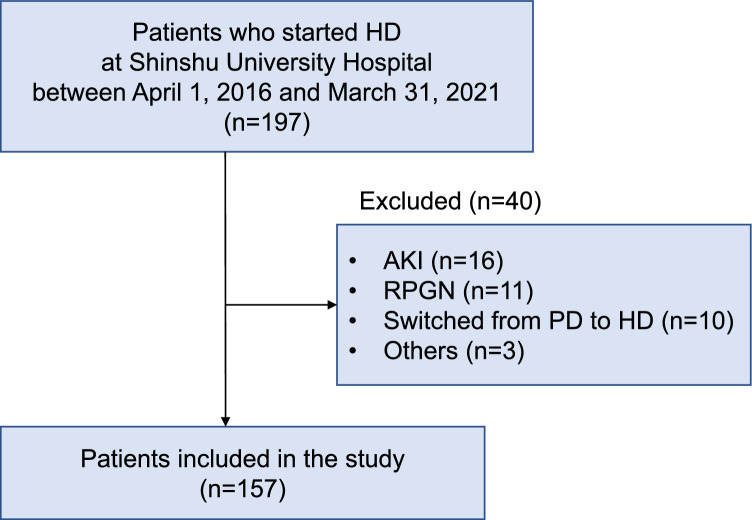
Study flowchart. *AKI* acute kidney injury, *HD* hemodialysis, *PD* peritoneal dialysis, *RPGN* rapidly progressive glomerulonephritis

A retrospective cohort study was conducted including 197 patients with CKD who commenced HD at Shinshu University Hospital between April 1, 2016, and March 31, 2021 (Fig. 1). Patients: aged < 18 years, who developed AKI, with rapidly progressive glomerulonephritis (RPGN), who shifted from peritoneal dialysis (PD) to HD, and with several missing data were excluded. Patients who were emergently started on HD were included when their clinical course was consistent with CKD progression but not with AKI. Finally, 157 patients with CKD were included in this study.

All patients with a prepared VA started HD with it, whereas those without a prepared VA began HD with a temporary central venous catheter. The Institutional Review Board of the Ethics Committee at Shinshu University School of Medicine approved the study. The requirement for written informed consent was waived because of the retrospective nature of the study.

### Data collection

Baseline patient characteristics included the presence of a prepared VA, age, sex, body mass index (BMI), smoking history, type of VA, PS, cause of CKD, systemic complications, Charlson Comorbidity Index (CCI), and controlling nutritional status (CONUT) score. Serum albumin, urea nitrogen, serum creatinine, potassium, and hemoglobin levels were collected before the first HD session. Clinical outcomes, such as all-cause mortality as a prognosis, hospitalization expenses at the time of planned VA creation and HD initiation, rate of home discharge, and duration of hospitalization, were investigated. Hospitalization expenses included all costs related to meals, inspections, and surgery. In patients who underwent planned VA creation and had a prepared VA, hospitalization expenses at the times of VA creation, VA maintenance, such as percutaneous transluminal angioplasty, and HD initiation, were combined and evaluated. We followed up the patients from HD initiation until death or March 31, 2022, whichever came first. Patients lost to follow-up were considered “censored” on their final hospital visit and included in the study. In patients without VA creation before HD initiation, we collected data on the reasons for the lack of VA creation.

### Definitions

The clinician determined HD initiation by considering kidney dysfunction severity, body fluid abnormalities, clinical symptoms, and the impact on daily functioning. Based on the Kidney Disease Improving Global Outcomes (KDIGO) guidelines [10], emergent HD initiation was defined as cases wherein HD must be initiated immediately or < 48 h after presentation to correct life-threatening manifestations. As the mean age of patients with HD in Japan is almost 70 years [11], we defined patients aged ≥ 70 and < 70 years as elderly and non-elderly, respectively. The causes of CKD were categorized as diabetic nephropathy, chronic glomerulonephritis, nephrosclerosis, and others based on medical records. Information on complications was also collected from medical records. In the current study, patients with a CCI score of ≥ 5 points were defined as those with a high mortality risk. Patients with a CONUT score of ≥ 5 points were classified as those with severe malnutrition.

### Statistical analyses

Categorical data were compared using the Chi-square test. Normally and non-normally distributed variables were expressed as means ± standard deviation and median (interquartile range). Student’s t-test and the Mann–Whitney U test were used to compare normally and non-normally distributed data, respectively. First, we investigated the association between age [elderly (≥ 70) or not elderly (< 70)] and mortality using Cox hazards regression analysis. Then, patient survival was evaluated using the Kaplan–Meier method, and the log-rank test was applied to compare survival rates between patients with and without a prepared VA. Univariate and multivariate Cox regression analyses were performed to investigate the factors associated with all-cause mortality. Univariate and multivariate logistic regression analyses were performed to investigate factors associated with hospitalization expenses. Using multivariate analyses, the presence of a prepared VA was adjusted for potential confounding factors, including sex, nutritional status (CONUT score), comorbidity (CCI), and PS based on their clinical significance as confounders of all-cause mortality. Owing to the limited number of outcomes using the multivariate analysis, two explanatory variables were included in this study. Hospitalization expenses were analyzed by dividing patients into two groups based on a median value of 1.3 million Japanese yen. We also investigated the interaction between “age” and “presence of prepared VA” with regard to mortality and high hospitalization expenses. The details of other analyses were described in Online Resource 1 (supplementary methods). All analyses were two-tailed, and statistical significance was set at P < 0.05. EZR (Saitama Medical Center, Jichi Medical University, Saitama, Japan), a graphical user interface for R (The R Foundation for Statistical Computing, Vienna, Austria), was used for all analyses.

## Results

### Baseline characteristics of study participants and comparison between patients with and without a prepared VA

Of the 157 patients, 116 (73.8%) had a prepared VA (Table 1). The median age at HD initiation was 69 years; 104 patients (66.2%) were men. Diabetic nephropathy was the most common cause of ESKD (42.7%). Among all patients, 90 (57.3%) had a CCI of ≥ 5 points and 94 (60.0%) had a CONUT score of ≥ 5 points. Between patients with and without a prepared VA, age, AVG creation rate, tunneled cuffed catheter insertion rate, emergent HD initiation, incidence of diabetes mellitus, high CCI, high CONUT score, serum albumin level, serum potassium level, duration of hospitalization at HD initiation, and hospitalization expenses were significantly lower in patients with a prepared VA (Table 1). Additionally, the AVF creation rate, incidence of glomerulonephritis as a cause of CKD, and rate of home discharge after HD initiation were significantly higher in patients with a prepared VA. The incidence of mortality was significantly higher among patients without a prepared VA.

**Table 1:** Background characteristics of all participants

	All (n = 157)	Prepared VA ( +) (n = 116)	Prepared VA (-) (n = 41)	P-value
Age	69.0 (59.0–79.0)	68.0 (55.0–78.2)	72.0 (65.0–80.0)	0.044
male (n,%)	104 (66.2)	80 (69.0)	24 (58.5)	0.25
BMI (kg/m^2^)	21.8 (19.7–24.7)	22.0 (20.1–24.8)	21.0 (19.0–22.7)	0.09
smoking history (n,%)	86 (54.7)	66 (56.9)	20 (48.8)	0.46
Types of VA (finally created)				
Arteriovenous fistulas (n,%)	146 (93.0)	113 (97.3)	33 (80.5)	< 0.001
Arteriovenous grafts (n,%)	4 (2.5)	1 (0.9)	3 (7.3)	0.02
Subcutaneously fixed superficial artery (n,%)	1 (0.6)	1 (0.9)	0 (0.0)	0.55
Tunneled cuffed catheters (n,%)	6 (3.9)	1 (0.9)	5 (12.2)	0.001
eGFR at the time of VA creation (mL/min/1.73m^2^)	–	7.2 (5.9–8.7)	–	
Period from VA creation to HD initiation (month)	–	2.4 (1.1–5.0)	–	
Emergent HD initiation (n,%)	79 (50.3)	38 (32.7)	41 (100.0)	< 0.001
PS				
0	115 (73.2)	90 (77.6)	25 (61.0)	0.06
1–4	42 (26.8)	26 (22.4)	16(39.0)	
Cause of CKD				
Diabetic nephropathy (n,%)	67 (42.7)	48 (41.4)	20 (48.8)	0.46
Nephrosclerosis (n,%)	8 (5.1)	9 (7.8)	5 (12.2)	0.52
Glomerulonephritis (n,%)	21 (13.4)	17 (14.7)	1 (2.4)	0.044
Others (n,%)	26 (16.5)	18 (15.5)	3 (7.3)	0.28
Unknown (n,%)	35 (22.3)	24 (20.7)	12 (29.3)	0.28
Complication				
Diabetes mellitus (n,%)	87 (55.4)	58 (50.0)	29 (70.7)	0.028
Coronary artery disease (n,%)	24 (15.2)	15 (12.9)	9 (22.0)	0.20
Cerebrovascular disease (n,%)	27 (17.1)	20 (17.2)	7 (17.1)	0.99
Peripheral vascular disease (n,%)	25 (15.9)	19 (16.4)	6 (14.6)	1.01
Dementia (n,%)	9 (5.7)	4 (3.4)	5 (12.2)	0.05
CCI, points (0–)				
0–4	67 (42.7)	56 (48.3)	11 (26.8)	0.018
≥ 5	90 (57.3)	60 (51.7)	30 (73.2)	
CONUT, points (0–)				
0 ~ 4	63 (40.0)	51 (44.0)	12 (29.3)	0.13
≥ 5	94 (60.0)	65 (56.0)	29 (70.7)	
Laboratory data				
Alb (g/dL)	3.1 (2.8–3.6)	2.8 (2.4–3.4)	3.2 (2.9–3.6)	0.002
BUN (mg/dL)	85.4 (68.0–99.4)	82.5 (66.4–104.0)	83.2 (70.1–97.7)	0.64
Cre (mg/dL)	8.1 (6.3–9.2)	7.6 (6.0–8.9)	8.1 (6.4–9.4)	0.13
eGFR (mL/min/1.73m^2^)	5.2 (4.4–6.4)	5.8 (4.7–7.2)	5.4 (4.3–7.1)	0.89
K (mmol/L)	4.5 (3.9–4.9)	4.2 (3.8–4.7)	4.6 (4.0–5.0)	0.038
Hb (g/dL)	9.3 (8.5–10.2)	9.1 (8.4–10.3)	9.5 (8.6–10.2)	0.63
Clinical outcomes				
Mortality (n,%)	30 (19.1)	16 (13.8)	14 (34.1)	0.010
Home discharge (n,%)	137 (87.2)	109 (94.0)	28 (68.3)	< 0.001
Duration of hospitalization (days)	15.0 (9.0–25.0)	12.0 (8.0–18.0)	31.0 (23.0–39.0)	< 0.001
Hospitalization expenses (JPY)	1,285,800 (964,610–1,636,330)	1,117,430 (880,476–1,396,847)	1,739,449 (1,384,900–2,208,830)	< 0.001

Categorical data were compared using the Chi-square test. Normally and non-normally distributed variables were expressed as means ± standard deviation and median (interquartile range). Student’s t-test and the Mann–Whitney U test were used to compare normally and non-normally distributed data, respectively. All analyses were two-tailed, and statistical significance was set at P < 0.05

*Alb* albumin, *BMI* body mass index, *BUN* blood urea nitrogen, *Cre* creatinine, *CCI* Charlson comorbidity index, *CKD* chronic kidney disease, *CONUT* controlling nutritional status, *eGFR* estimated glomerular filtration rate, *HD* hemodialysis, *Hb* hemoglobin, *K* potassium, *PS* performance status, *VA* vascular access

### Baseline characteristics between patients with and without a prepared VA in the elderly and non-elderly groups

Of the 76 elderly patients with and without a prepared VA at HD initiation, 52 (68.4%) had a prepared VA. The PS, CCI score, and CONUT score did not differ between the two groups; however, the AVF creation rate was significantly higher, and emergent HD initiation, hospitalization duration, and hospitalization expenses during HD initiation were significantly lower in patients with a prepared VA (Table 2). In elderly patients, the incidence of mortality was comparable between those with a prepared VA and those without a prepared VA (Table 2). Concerning non-elderly patients at the time of HD initiation, age, high CCI and CONUT scores, emergent HD initiation, duration of hospitalization, and hospitalization expenses during initiation of HD were significantly lower in patients with a prepared VA (Table 3). In non-elderly patients, the incidence of mortality was significantly higher among those without a prepared VA. Additionally, the serum albumin level, potassium level, and rate of home discharge after HD initiation were significantly higher in patients with a prepared VA (Table 3). Cardiovascular disease was the most common cause in both elderly and non-elderly patients (tables, Online Resources 2 and 3). However, the exact cause of mortality was unknown in 10 of 30 patients (tables, Online Resources 2 and 3).

**Table 2:** Background characteristics of elderly patients (≥ 70 years)

	All	Prepared VA ( +)	Prepared VA (-)	P-value
	(N = 76)	(n = 52)	(n = 24)	
Age	79.0 (74.0–83.0)	79.0 (74.5–85.5)	79.0 (74.7–82.2)	0.94
male (n,%)	52 (68.4)	38 (73.1)	14 (58.3)	0.28
BMI (kg/m^2^)	21.5 (20.5–23.3)	21.0 (19.9–22.6)	22.0 (20.3–23.9)	0.26
Smoking history (n,%)	41 (53.9)	32 (61.5)	9 (37.5)	0.08
Types of VA (finally created)				
Arteriovenous fistulas (n,%)	67 (88.2)	50 (96.2)	17 (70.9)	0.001
Arteriovenous grafts (n,%)	3 (3.9)	1 (1.9)	2 (8.3)	0.18
Subcutaneously fixed superficial artery (n,%)	1 (1.3)	1 (1.9)	0 (0.0)	0.49
Tunneled cuffed catheters (n,%)	5 (6.6)	0 (0.0)	5 (20.8)	< 0.001
eGFR at the time of VA creation (mL/min/1.73m^2^)	–	7.2 (6.0–9.1)	–	
Period from VA creation to HD initiation (month)	–	2.1 (0.9–5.0)	–	
Emergent HD initiation (n,%)	43 (56.5)	19 (38.0)	24 (100.0)	< 0.001
PS				
0	43 (56.6)	31 (59.6)	12 (50.0)	0.46
1–4	33 (43.4)	21 (40.4)	12 (50.0)	
Cause of CKD				
Diabetic nephropathy (n,%)	32 (42.1)	21 (40.4)	11 (45.8)	0.80
Nephrosclerosis (n,%)	5 (6.6)	3 (5.8)	2 (8.3)	0.65
Glomerulonephritis (n,%)	9 (11.8)	9 (17.3)	0 (0.0)	0.040
Others (n,%)	11 (14.5)	8 (15.4)	3 (12.5)	1.00
Unknown (n,%)	19 (25.0)	11 (21.2)	8 (33.3)	0.27
Complication				
Diabetes mellitus (n,%)	43 (56.6)	27 (48.1)	16 (16.0)	0.32
Coronary artery disease (n,%)	15 (19.7)	9 (17.3)	6 (25.0)	0.54
Cerebrovascular disease (n,%)	18 (23.7)	15 (71.2)	3 (12.5)	0.15
Peripheral vascular disease (n,%)	20 (26.3)	15 (28.8)	5 (20.8)	0.58
Dementia (n,%)	9 (11.8)	4 (7.7)	5 (20.8)	0.13
CCI, points (0–)				
0–4	23 (30.3)	17 (32.7)	6 (25.0)	0.59
≥ 5	53 (69.7)	35 (67.3)	18 (75.0)	
CONUT, points (0–)				
0 ~ 4	21 (27.6)	15 (28.8)	6 (25.0)	0.79
≥ 5	55 (72.4)	37 (71.2)	18 (75.0)	
Laboratory data				
Alb (g/dL)	3.0 (2.7–3.5)	3.1 (2.8–3.5)	2.8 (2.4–3.3)	0.11
BUN (mg/dL)	83.6 (69.3–99.4)	84.0 (76.4–99.4)	82.4 (65.8–100.0)	0.79
Cre (mg/dL)	7.2 (6.2–8.8)	7.3 (6.2–9.1)	6.7 (5.7–8.2)	0.33
eGFR (mL/min/1.73m^2^)	5.6 (4.5–7.2)	5.5 (4.6–7.2)	5.7 (4.7–7.1)	0.70
K (mmol/L)	4.3 (3.9–4.9)	4.5 (4.0–4.9)	3.9 (3.5–4.4)	0.010
Hb (g/dL)	9.4 (8.6–10.3)	9.4 (8.6–10.3)	9.5 (8.7–10.2)	0.86
Clinical outcomes				
Mortality (n,%)	22 (28.9)	13 (25.0)	9 (37.5)	0.29
Home discharge (n,%)	63 (82.9)	46 (88.5)	17 (70.8)	0.09
Duration of hospitalization (days)	18.0 (10.0–28.0)	13.0 (9.0–20.0)	32.3 (23.0–39.0)	< 0.001
Hospitalization expenses (JPY)	1,384,900 (1,059,689–1,721,175)	1,272,330 (972,340–1,518,553)	1,730,307 (1,388,425–2,094,667)	< 0.001

Categorical data were compared using the Chi-square test. Normally and non-normally distributed variables were expressed as means ± standard deviation and median (interquartile range). Student’s t-test and the Mann–Whitney U test were used to compare normally and non-normally distributed data, respectively. All analyses were two-tailed, and statistical significance was set at P < 0.05

*Alb* albumin, *BMI* body mass index, *BUN* blood urea nitrogen, *Cre* creatinine, *CCI* Charlson comorbidity index, *CKD* chronic kidney disease, *CONUT* controlling nutritional status, *eGFR* estimated glomerular filtration rate, *HD* hemodialysis, *Hb* hemoglobin, *K* potassium, *PS* performance status, *VA* vascular access

**Table 3:** Background characteristics of non-elderly patients (< 70 years)

	All N = 81)	Prepared VA ( +) (n = 64)	Prepared VA (-) (n = 17)	P-value
Age	60.0 (50.0–64.0)	58.0 (48.0–63.2)	63.0 (57.0–68.0)	0.048
male (n,%)	52 (64.2)	42 (65.6)	10 (58.8)	0.77
BMI (kg/m^2^)	22.3 (19.3–26.1)	22.1 (20.1–26.3)	20.8 (17.8–25.4)	0.22
smoking history (n,%)	45 (55.6)	34 (53.1)	11 (64.7)	0.42
Types of VA (finally created)				
Arteriovenous fistulas (n,%)	79 (97.6)	63 (98.4)	16 (94.1)	0.31
Arteriovenous grafts (n,%)	1 (1.2)	0 (0.0)	1 (5.9)	0.05
Subcutaneously fixed superficial artery (n,%)	0 (0.0)	0 (0.0)	0 (0.0)	
Tunneled cuffed catheters (n,%)	1 (1.2)	1 (1.6)	0 (0.0)	0.60
eGFR at the time of VA creation (mL/min/1.73m^2^)	-	7.2 (5.8–8.3)	-	
Period from VA creation to HD initiation (month)	-	3.1 (1.2–5.1)	-	
Emergent HD initiation (n,%)	36 (44.4)	19 (29.7)	17 (100.0)	< 0.001
PS				
0	72 (88.9)	59 (92.2)	13 (76.5)	0.08
1–4	9 (11.1)	5 (7.8)	4 (23.5)	
Cause of CKD				
Diabetic nephropathy (n,%)	35 (43.2)	27 (42.2)	8 (47.1)	0.78
Nephrosclerosis (n,%)	3 (3.7)	1 (1.6)	2 (11.8)	0.11
Glomerulonephritis (n,%)	12 (14.8)	10 (15.6)	2 (11.8)	1.00
Others (n,%)	14 (17.3)	13 (20.3)	1 (5.9)	0.28
Unknown (n,%)	17 (21.0)	13 (20.3)	4 (23.5)	0.74
Complication				
Diabetes mellitus (n,%)	44 (54.3)	31 (48.4)	13 (76.5)	0.05
Coronary artery disease (n,%)	9 (11.1)	6 (9.4)	3 (17.6)	0.38
Cerebrovascular disease (n,%)	9 (11.1)	5 (7.8)	4 (23.5)	0.08
Peripheral vascular disease (n,%)	5 (6.2)	4 (6.2)	1 (5.9)	0.58
Dementia (n,%)	0 (0.0)	0 (0.0)	0 (0.0)	1.00
CCI, points (0–)				
0–4	44 (54.3)	39 (60.9)	5 (29.4)	0.028
≥ 5	37 (45.7)	25 (39.1)	12 (70.6)	
CONUT, points (0–)				
0 ~ 4	42 (51.8)	36 (56.2)	6 (35.3)	0.17
≥ 5	39 (48.2)	28 (43.8)	11 (64.7)	
Laboratory data				
Alb (g/dL)	3.2 (2.8–3.7)	3.4 (2.9–3.8)	2.6 (2.2–3.7)	0.020
BUN (mg/dL)	81.8 (67.3–98.3)	81.6 (67.6–95.3)	93.1 (67.3–106.1)	0.37
Cre (mg/dL)	8.5 (7.0–10.1)	8.59 (7.3–10.1)	8.4 (6.1–9.2)	0.57
eGFR (mL/min/1.73m^2^)	5.1 (3.9–6.4)	5.3 (4.3–6.4)	5.4 (4.3–7.1)	0.89
K (mmol/L)	4.6 (4.0–5.0)	4.7 (4.0–5.0)	4.5 (4.0–4.8)	0.010
Hb (g/dL)	9.4 (8.3–10.1)	9.5 (8.5–10.1)	8.8 (7.9–10.7)	0.33
Clinical outcomes				
Mortality (n,%)	8 (9.9)	3 (4.7)	5 (29.4)	0.009
Home discharge (n,%)	74 (91.4)	63 (98.4)	11 (64.7)	< 0.001
Duration of hospitalization (days)	14.0 (9.0–20.0)	12.0 (8.0–17.2)	30.0 (22.0–38.0)	< 0.001
Hospitalization expenses (JPY)	1,123,185 (895,296–1,479,183)	1,023,050 (863,119–1,306,610)	2,007,466 (1,355,326–2,998,550)	< 0.001

Categorical data were compared using the Chi-square test. Normally and non-normally distributed variables were expressed as means ± standard deviation and median (interquartile range). Student’s t-test and the Mann–Whitney U test were used to compare normally and non-normally distributed data, respectively. All analyses were two-tailed, and statistical significance was set at P < 0.05

*Alb* albumin, *BMI* body mass index, *BUN* blood urea nitrogen, *Cre* creatinine, *CCI* Charlson comorbidity index, *CKD* chronic kidney disease, *CONUT* controlling nutritional status, *eGFR* estimated glomerular filtration rate, *HD* hemodialysis, *Hb* hemoglobin, *K* potassium, *PS* performance status, *VA* vascular access

### Survival rates between patients with and without a prepared VA in the non-elderly and elderly groups

**Fig. 2:**
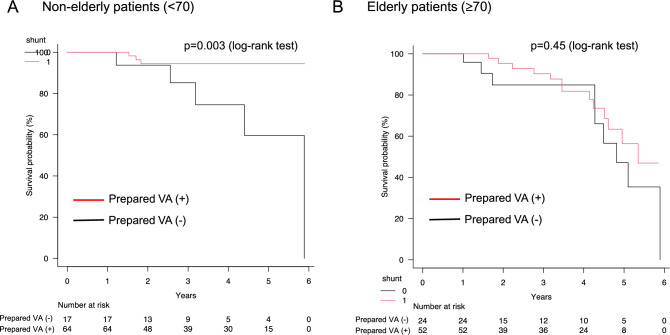
Comparison of survival rates between patients with and without a prepared VA divided into non-elderly and elderly groups. The survival rate was evaluated using the Kaplan–Meier method and the log-rank test. **A** Comparison of survival rates among non-elderly patients with CKDG5 with and without a prepared VA. **B** Comparison of survival rates among elderly patients with CKDG5 with and without a prepared VA. *VA* vascular access

In the non-elderly group, the survival rate was significantly higher in patients with a prepared VA (log-rank test, P < 0.05) (Fig. 2A). However, the survival rate decreased in a time-dependent manner regardless of the presence of a prepared VA in the elderly group. The survival rates did not significantly differ between the two groups (log-rank test, P = 0.45) (Fig. 2B).

### Association of a prepared VA with all-cause mortality and hospitalization expenses

Cox proportional hazards regression analysis demonstrated that age [elderly (≥ 70) or not elderly (< 70)] was significantly associated with mortality in the crude model (P = 0.007; hazard ratio, 3.07; 95% confidence interval, 1.37–6.91; not presented in the Table). Cox proportional hazards regression analyses were conducted to determine the association between the presence of a prepared VA and all-cause mortality. In non-elderly patients, the presence of a prepared VA was significantly associated with all-cause mortality independent of sex, nutritional status (CONUT score), comorbidity (CCI), and PS (Table 4); this was not observed in elderly patients. Meanwhile, PS was significantly associated with all-cause mortality in elderly patients. Using logistic regression analyses, the association between a prepared VA and hospitalization expenses was determined. The presence of a prepared VA was significantly associated with high hospitalization expenses in both non-elderly and elderly patients after adjusting for sex, nutritional status (CONUT score), comorbidity (CCI), and PS (Table 5). Using the interaction analyses, although there was no significant association between the interaction term “age: presence of prepared VA” and all-cause mortality using Cox hazards regression analysis (table, Online Resource 4) in elderly and non-elderly patients, we found a significant association between the interaction term “age: presence of prepared VA” and all-cause mortality in all patients. In addition, the interaction term “age: presence of prepared VA” did not show a significant association with high hospitalization expenses using logistic regression analysis in any of the three groups of patients (all patients, elderly, and non-elderly) (table, Online Resource 5).

**Table 4:** Cox hazards regression analyses of factors associated with all-cause mortality

	Elderly (≥ 70)			Non-elderly (< 70)		
	HR	95% CI	P-value	HR	95% CI	P-value
Model 1						
Presence of prepared VA (unadjusted)	0.71	0.29–1.73	0.46	0.17	0.03–0.72	0.01
Model 2						
Presence of prepared VA	0.63	0.25–1.56	0.32	0.17	0.03–0.74	0.01
male	2.03	0.76–5.37	0.15	1.49	0.28–7.74	0.63
Model 3						
Presence of prepared VA	0.71	0.29–1.74	0.45	0.23	0.06–0.83	0.02
CCI ≥ 5	1.03	0.39–2.69	0.95	7.91	1.46–42.82	0.01
Model 4						
Presence of prepared VA	0.71	0.29–1.72	0.45	0.21	0.05–0.94	0.04
CONUT ≥ 5	0.91	0.35–2.38	0.85	4.37	0.51–37.42	0.17
Model 5						
Presence of prepared VA	0.94	0.38–2.31	0.90	0.28	0.06–0.87	0.04
PS 1–4	4.25	1.61–11.13	0.003	7.75	1.60–37.44	0.01

Univariate and multivariate Cox hazards regression analyses were performed to investigate factors associated with all-cause mortality. In multivariate analyses, the presence of a prepared VA was adjusted for potential confounding factors, including sex, nutritional status (CONUT score), comorbidity (CCI), and PS based on their clinical significance as confounders of all-cause mortality. Statistical significance was set at P < 0.05

*CCI* Charlson comorbidity index, *CONUT* controlling nutritional status, *PS* performance status, *VA* vascular access

**Table 5:** Logistic regression analyses of factors associated with high hospitalization expenses (> 1,300,000 JPY)

	Elderly (≥ 70)			Non-elderly (< 70)		
	OR	95% CI	P-value	OR	95% CI	P-value
Model 1						
Presence of prepared VA (unadjusted)	0.13	0.03–0.52	0.003	0.07	0.02–0.29	< 0.001
Model 2						
Presence of prepared VA	0.14	0.03–0.53	< 0.001	0.05	0.01–0.23	< 0.001
Male	0.88	0.29–2.67	0.82	4.24	1.09–16.60	0.04
Model 3						
Presence of prepared VA	0.13	0.03–0.51	< 0.001	0.05	0.01–0.23	< 0.001
CCI ≥ 5	0.87	0.29–2.61	0.81	2.96	0.99–8.82	0.05
Model 4						
Presence of prepared VA	0.15	0.04–0.58	0.006	0.10	0.02–0.42	< 0.001
CONUT ≥ 5	1.39	0.46–4.15	0.56	2.23	0.83–6.01	0.11
Model 5						
Presence of prepared VA	0.14	0.03–0.54	< 0.001	0.08	0.02–0.33	< 0.001
PS 1–4	1.86	0.65–5.35	0.25	4.21	0.65–27.33	0.13

Univariate and multivariate logistic regression analyses were performed to investigate factors associated with hospitalization expenses. In multivariate analyses, the presence of a prepared VA was adjusted for potential confounding factors, including sex, nutritional status (CONUT score), comorbidity (CCI), and PS based on their clinical significance as confounders of all-cause mortality. Statistical significance was set at P < 0.05

*CCI* Charlson comorbidity index, *CONUT* controlling nutritional status, *PS* performance status, *VA* vascular access

### Subgroup analyses excluding patients whose final VA was a superficial artery or tunneled cuffed catheter

Since most patients wherein the final VA was a superficial artery or tunneled cuffed catheter had chronic heart failure with poor vascular conditions, their prognosis was predictably poor. Therefore, we performed subgroup analysis that excluded these patients. In patients wherein the final VA was an AVF or AVG, a prepared VA was associated with all-cause mortality only in non-elderly patients (log-rank test, P = 0.002) (figure, Online Resource 6). Regarding hospitalization expenses, a prepared VA was a significant factor in non-elderly and elderly patients (table, Online Resource 7).

### Subgroup analyses of patients who underwent emergency HD

Since patients who underwent emergency HD might have higher mortality, potentially concealing the impact of a prepared VA on mortality, we conducted subgroup analysis after dividing all patients into those who underwent emergency HD and others who did not. Survival rates in patients who underwent emergency HD were comparable between non-elderly and elderly patients with or without a prepared VA (log-rank test, P = 0.053 and P = 0.85, respectively) (figure, Online Resource 8). Regarding the hospitalization expenses in patients who underwent emergent HD, univariate logistic regression analyses revealed that a prepared VA was not associated with high hospitalization expenses in both non-elderly and elderly patients (table, Online Resource 9). We could not perform a statistical analysis of data of patients who did not require emergency HD as all, except one, had a prepared VA.

### Reasons for lack of VA creation before HD initiation

**Fig. 3:**
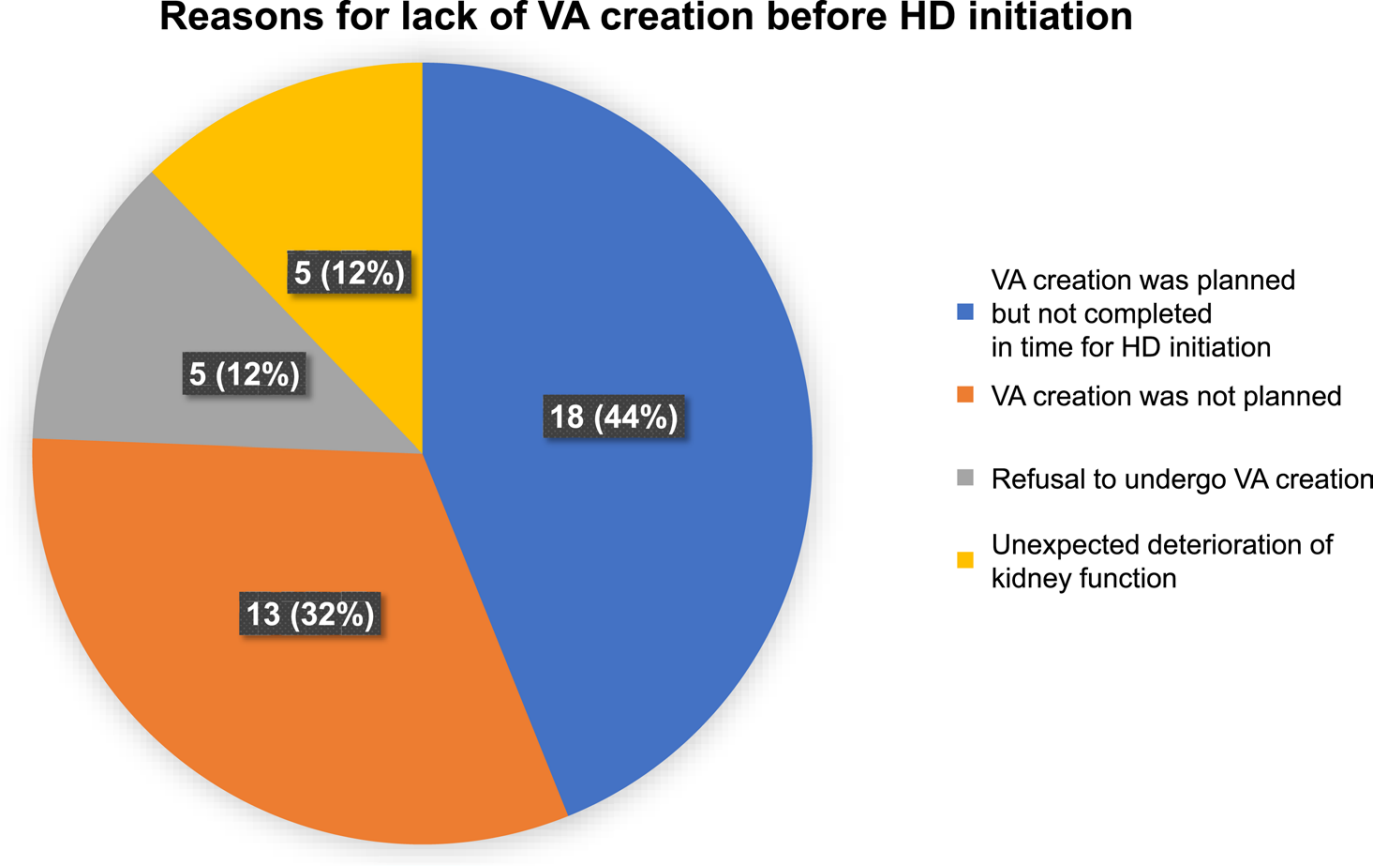
Reasons for lack of VA creation before HD initiation. The pie chart shows the different reasons for the lack of VA creation before HD initiation. *HD* hemodialysis, *VA* vascular access

Among the patients without VA creation before HD initiation, VA creation was planned but not completed in time for HD initiation for 44% of patients, whereas it was not planned for 32% of patients (Fig. 3). In addition, 12% of patients refused to undergo VA creation while the remaining 12% showed unexpected deterioration in kidney function.

## Discussion

A prepared VA showed several benefits, including lower mortality rates and hospitalization costs; shorter hospital stays; and higher home discharge rates. Additionally, a prepared VA was associated with lower hospitalization expenses regardless of age and clinical factors.

Among non-elderly patients, the benefit of planned VA creation appeared obvious, and our results are consistent with those of previous research, indicating a correlation between suboptimal dialysis initiation and early mortality [12–14]. Unplanned VA creation was reportedly associated with increased mortality within 4 months after starting dialysis, along with heightened medical expenses in Japan [15]. Regarding the differences between this study and previous reports, ours excluded patients with AKI and stratified patients with CKDG5 based on age. AKI is an acute condition that does not allow for planned VA creation and increases the risk of subsequent mortality and medical expenses. Since patients with AKI were excluded in this study, the impact of planned VA creation on patient mortality became clear. Pre-dialysis education is crucial for planned VA creation before HD initiation as it leads to successful HD initiation and decreases mortality risk [16–18].

In elderly patients, a prepared VA was not significantly associated with all-cause mortality, even after adjusting for sex, PS, complications, or nutritional status. Emergent HD is also significantly associated with mortality in both non-elderly and elderly patients [12–14]. This study demonstrated that the emergent HD rate was significantly lower regardless of patient age with a prepared VA; however, an obvious association between all-cause mortality and a prepared VA was not observed in elderly patients, suggesting a stronger influence of other factors. This study demonstrated that poor PS was the only risk factor for all-cause mortality in elderly patients with CKD. In Japan, the age of patients with HD has increased remarkably. The Annual Dialysis Data Report for 2022 showed that the average age of patients on HD increased yearly (71.42 years; 70.76 in men, 72.92 in women). The age group with the highest HD initiation rate was 70–74 years in men and 80–84 years in women, indicating that Japanese patients with HD are the oldest worldwide. The advanced age of Japanese patients during HD initiation may lead to a decline in PS, significantly affecting their mortality and negating the benefit of planned VA creation in elderly patients. Recently, conservative kidney management (CKM), a non-dialysis option has focused on palliative care, is a feasible option that does not affect life expectancy or quality of life in elderly patients with CKD or those with multiple complications [19]. Considering future treatment options, including CKM, in elderly CKD patients, considering the patient’s general condition and predicted prognosis of managing these patients is essential. This study demonstrated that planned VA creation decreases medical expenses and duration of hospitalization, even in elderly patients with CKD. As the general condition of elderly patients with CKD varies, planned VA creation may be recommended for elderly patients with CKD without poor PS. A large-scale study is essential to validate these results and provide further insights.

The type of VA is associated with prognosis in patients [20–22]. Shunt VA, including AVF and AVG, is associated with a better prognosis than a central venous catheter (non-shunt VA) in elderly patients with CKD [23]. Japan’s AVF and AVG creation rates are higher than those in European countries and the U.S. [24]. This indicates that the difference in the types of VA between Japan and other countries might have affected the results. Therefore, we performed subgroup analyses that only included patients with an AVF or AVG. Results showed that all-cause mortality did not differ between elderly patients with and without a prepared AVF/AVG. As the number of patients with a non-shunt VA was small in our study, an analysis targeting patients with a non-shunt VA was complex.

Hospitalization expenses during HD initiation are lower in patients with a prepared VA [15, 25]; the results of this study strongly support these findings. The difference in medical expenses influenced by the lack of a prepared VA may be attributed to the utilization of a central venous catheter and longer hospitalization. However, this study indicated that all-cause mortality and hospitalization expenses were comparable in patients requiring emergent HD regardless of the presence of a prepared VA and age. These findings suggest that emergent HD is one of the strongest prognostic factors and may conceal the benefits of planned VA creation.

Regarding the timing of VA creation in our patients, the median estimated glomerular filtration rate (eGFR) at the time of VA creation was 7.2 (5.9–8.7) mL/min/1.73 m^2^, and the median period from VA creation to HD initiation was 2.4 (1.1–5.0) months. In Japan, considering VA creation when eGFR falls below 15 mL/min/1.73 m^2^ is recommended, ideally completing it 2–3 weeks before HD initiation [26]. Most patients in this study met this recommendation. Prepared VA may contribute to heart failure by the prolonged period after VA creation and increased amount of blood flow of VA. No patients were hospitalized for heart failure between VA creation and HD initiation. However, acknowledging the potential for fluid overload-induced heart failure in patients initiating HD is essential, which might be exacerbated by the presence of VA. In addition, prepared VA may cause the functional failure of VA between VA creation and HD initiation. In this study, 10 patients required reoperation or percutaneous transluminal angioplasty owing to VA issues before HD initiation.

With regard to reasons why VA was not created before HD initiation in some patients, the unexpected deterioration in kidney function in 12% of patients may, by itself, have been associated with mortality because underlying disease can affect the prognosis. In the remaining 88% of patients, it is considered that there was no direct association with mortality. Although knowledge about the cause of mortality would be useful, we could not determine the exact reason for 10 out of 30 patients.

This study has some limitations. The study was conducted at a single center with a small sample size. The decision regarding whether to create a VA in patients with CKDG5 might differ in each center or among clinicians. Although we collected data regarding the exact cause of mortality, it was unknown for 10 out of 30 patients, because most patients continued HD at other hospitals after initiating the procedure at our hospital. In addition, because of the small number of mortality outcomes and many undefined causes of mortality, effectively comparing the causes of mortality between elderly and non-elderly patients was difficult. Furthermore, confounding factors may not have been adequately adjusted for using the multivariate analyses. Particularly, we could not include all factors in the model at the same time because of the small number of outcomes. This finding, that an interaction between age and the presence of VA was observed in relation to mortality across all patients, supports the appropriateness of stratifying patients by age at least regarding the analyses for mortality. However, the cutoff age of “70 years” in the current study was based on the fact that the mean age of patients on HD in Japan is almost 70 years. Therefore, the cutoff age of “70 years” does not have any statistical basis linked to our dataset. Therefore, the results might be changed based on the definition of age of “elderly” and we could not precisely determine from which age the effectiveness of VA creation could differ. Large-scale, prospective studies are warranted to overcome all these limitations. Next, the present study exclusively focused on medical expenses within the Japanese healthcare system, which operates under a universal insurance model. Given that healthcare systems vary widely among countries, the results of this study might not be directly applicable to other countries with different cost structures and access to healthcare.

## Conclusion

This study verified that a VA created following VA creation planning offered various benefits, including lower mortality rates and hospitalization costs; shorter hospital stays; and higher home discharge rates. Planned VA creation was significantly associated with lower hospitalization expenses, irrespective of age. From a medical–economic perspective, planned VA creation might benefit all patients with CKDG5. From a prognostic perspective, selecting the method of renal replacement therapy and VA creation in elderly patients must consider their general condition as well as their preferences. An individualized approach that allows sufficient time would be more appropriate, considering the unique circumstances and preferences of elderly patients with CKD.

## Ethical approval

All procedures performed in studies involving human participants were in accordance with the ethical standards of the institutional committee at which the studies were conducted and with the 1964 Helsinki Declaration and its later amendments or comparable ethical standards. The Institutional Review Board of Shinshu University School of Medicine approved the study (2022–5538).

## Informed consent

The requirement for written informed consent was waived due to the retrospective nature of this study.

## Supplementary Information

Below is the link to the electronic supplementary material.

Supplementary file1 (DOCX 76 KB)

## Data Availability

The datasets generated and/or analyzed in the current study are available from the corresponding author upon reasonable request.

## Abbreviations

AKI: Acute kidney injury
AVF: Arterio-venous fistula
AVG: Arterio-venous graft
CCI: Charlson's comorbidity index
CKD: Chronic kidney disease
CONUT score: Controlling Nutrition Status score
ESKD: End stage kidney disease
HD: Hemodialysis
HR: Hazard ratio
VA: Vascular access

## References

[1] Keith DS, Nichols GA, Gullion CM, Brown JB, Smith DH. Longitudinal follow-up and outcomes among a population with chronic kidney disease in a large managed care organization. Arch Intern Med. 2004;164:659–63. 15037495 10.1001/archinte.164.6.659

[2] Nakamura K, Okamura T, Hayakawa T, et al. Chronic kidney disease is a risk factor for cardiovascular death in a community-based population in Japan NIPPON DATA. Circ J. 2006;70(90):954–9. 16864924 10.1253/circj.70.954

[3] Rhee CM, Kovesdy CP. Epidemiology: spotlight on CKD deaths-increasing mortality worldwide. Nat Rev Nephrol. 2015;11:199–200. 25734769 10.1038/nrneph.2015.25 PMC4379111

[4] Nakayama M, Sato T, Miyazaki M, et al. Increased risk of cardiovascular events and mortality among non-diabetic chronic kidney disease patients with hypertensive nephropathy: the Gonryo study. Hypertens Res. 2011;34:1106–10. 21796127 10.1038/hr.2011.96

[5] Ortega T, Ortega F, Diaz-Corte C, Rebollo P, Ma Baltar J, Alvarez-Grande J. The timely construction of arteriovenous fistulae: a key to reducing morbidity and mortality and to improving cost management. Nephrol Dial Transplant. 2005;20:598–603. 15647308 10.1093/ndt/gfh644

[6] Lorenzo V, Martn M, Rufino M, Hernández D Torres A, Ayus JC. Predialysis nephrologic care and a functioning arteriovenous fistula at entry are associated with better survival in incident hemodialysis patients: an observational cohort study. Am J Kidney Dis. 2004; 43:999–1007. 10.1053/j.ajkd.2004.02.012 15168379

[7] Astor BC, Eustace JA, Powe NR, Klag MJ, Fink NE, Coresh J. Type of vascular access and survival among incident hemodialysis patients: the Choices for Healthy Outcomes in Caring for ESRD (CHOICE) Study. J Am Soc Nephrol. 2005;16:1449–55. 15788468 10.1681/ASN.2004090748

[8] Wasse H, Speckman RA, McClellan WM. Arteriovenous fistula use is associated with lower cardiovascular mortality compared with catheter use among ESRD patients. Semin Dial. 2008;21:483–9. 18764794 10.1111/j.1525-139X.2008.00467.x PMC2692608

[9] Ng L, Chen F, Pisoni R, et al. Hospitalization risks related to vascular access type among incident US hemodialysis patients. Nephrol Dial Transplant. 2011;26:3659–66. 21372255 10.1093/ndt/gfr063

[10] De Ulíbarri J, González-Madroño A, de Villar NG, et al. CONUT : a tool for controlling nutritional status. First validation in a hospital population. Nutr Hosp. 2005; 20:38–45. 15762418

[11] Hanafusa N, Abe M, Joki N, et al. 2021 Annual dialysis data report, JSDT renal data registry. Journal of Japanese Society for Dialysis Therapy. 2022;55:665–723.

[12] Descamps C, Labeeuw M, Trolliet P, et al. Confounding factors for early death in incident end-stage renal disease patients: role of emergency dialysis start. Hemodial Int. 2011;15:23–9. 21223483 10.1111/j.1542-4758.2010.00513.x

[13] Couchoud C, Moranne O, Frimat L, Labeeuw M, Allot V, Stengel B. Associations between comorbidities, treatment choice and outcome in the elderly with end stage renal disease. Nephrol Dial Transplant. 2007;22:3246–54. 17616533 10.1093/ndt/gfm400

[14] Michel A, Pladys A, Bayat S, Couchoud C, Hannedouche T, Vigneau C. Deleterious effects of dialysis emergency start, insights from the French REIN registry. BMC Nephrol. 2018;19:233. 30223784 10.1186/s12882-018-1036-9 PMC6142323

[15] Shimizu Y, Nakata J, Yanagisawa N, et al. Emergent initiation of dialysis is related to an increase in both mortality and medical costs. Sci Rep. 2020;10:19638. 33184445 10.1038/s41598-020-76765-0 PMC7661714

[16] Marrón B, Ortiz A, de Sequera P, et al. Impact of end-stage renal disease care in planned dialysis start and type of renal replacement therapy - a Spanish multicentre experience. Nephrol Dial Transplant. 2006;21:51–5. 16825262 10.1093/ndt/gfl191

[17] Goldstein M, Yassa T, Dacouris N, McFarlane P. Nultidisciplinary predialysis care and morbidity and mortality of patients on dialysis. Am J Kidney Dis. 2004;44:706–14. 15384022

[18] Hammelgarn B, Manns B, Zhang J, et al. Association between Multidisciplinary Care and Survival for Elderly Patients with Chronic Kidney Disease. J Am Soc Nephrol. 2007;18:933–99. 10.1681/ASN.2006080860 17267742

[19] Raghavan D, Holley J. Conservative care of the elderly CKD patient: a practical guide. Adv Chronic Kidney Dis. 2016;23:51–6. 26709063 10.1053/j.ackd.2015.08.003

[20] Hicks C, Canner J, Arhuidese I, et al. Mortality benefits of different hemodialysis access types are age dependent. J Vasc Surg. 2015;61:449–56. 25175630 10.1016/j.jvs.2014.07.091

[21] Zhang J, Al-Jaishi A, Na Y, de Sa E, Moist LM. Association between vascular access type and patient mortality among elderly patients on hemodialysis in Canada. Hemodial Int. 2014;18:616–24. 24636659 10.1111/hdi.12151

[22] Desilva R, Patibandla B, Vin Y, et al. Fistula first is not always the best strategy for the elderly. J Am Soc Nephrol. 2013;24:1297–304. 23813216 10.1681/ASN.2012060632 PMC3736704

[23] Arhuidese I, Orandi B, Nejim B, Malas M. Utilization, patency and complications associated with vascular access for hemodialysis in the United States. J Vasc Surg. 2018;68:1166–77. 30244924 10.1016/j.jvs.2018.01.049

[24] Robinson BM, Akizawa T, Jager J, Kerr PG, Saran R, Pisoni RL. Factors affecting outcomes in patients reaching end-stage kidney disease worldwide: differences in access to renal replacement therapy, modality use, and hemodialysis practices. Lancet. 2016;388:294–306. 27226132 10.1016/S0140-6736(16)30448-2 PMC6563337

[25] Canadian Institute for Health Information. 2007 CORR Report - Treatment of End-Stage Organ Failure in Canada 1996 to 2005. Ottawa, CIHI; 2008

[26] Kukita K, Ohira S, Amano I, et al. 2011 update Japanese Society for Dialysis Therapy Guidelines of Vascular Access Construction and Repair for Chronic Hemodialysis. Ther Apher Dial. 2015;19:1–39. 25817931 10.1111/1744-9987.12296

