# Supplementary material for: Impact of prepared vascular access on mortality and medical expenses in elderly and non-elderly Japanese patients with chronic kidney disease stage G5: a retrospective cohort study
Source: Clin Exp Nephrol. 2025 Mar 18;29(8):1063–74. doi: 10.1007/s10157-025-02654-3 (PMC12331761; doi:10.1007/s10157-025-02654-3)
Supplement: Supplementary file 1 — Supplementary file1 (DOCX 76 KB) [file 10157_2025_2654_MOESM1_ESM.docx]

**Electronic Supplementary Material**

**Impact of Prepared Vascular Access on Mortality and Medical Expenses in Elderly and Non-Elderly Japanese Patients with Chronic Kidney Disease Stage G5: A Retrospective Cohort Study**

Takayuki Nimura^1^, Makoto Harada^1^, Daiki Aomura^1^, Kosuke Yamaka^1^, Koji Hashimoto^1^, Yuji Kamijo^1^

^1^Department of Nephrology, Shinshu University School of Medicine, 3-1-1, Asahi, Matsumoto, 390–8621, Japan

**Corresponding author**

Yuji Kamijo, M.D., Ph.D.

E-mail: [yujibeat@shinshu-u.ac.jp](mailto:yujibeat@shinshu-u.ac.jp)

**Online Resource 1. Supplementary methods**

***Definitions***

Diabetes was defined as a glycated hemoglobin level >6.5% and/or prescription of glucose-lowering drugs, including insulin. Coronary, cerebral, and peripheral artery diseases were defined as a history of treatment. The controlling nutritional status (CONUT) score was calculated based on serum albumin, total cholesterol levels, and lymphocyte counts [10]. The Charlson Comorbidity Index (CCI) is a validated method for classifying comorbidities based on the International Classification of Diseases diagnosis codes. Each comorbidity category was assigned a weight ranging between 1 and 6, with cumulative weights yielding a single comorbidity score. A score of 0 indicated the absence of any comorbidities, whereas a higher score indicated a greater likelihood of adverse outcomes.

***Statistical analyses***

Although the vascular access (VA) type was determined after patient entry and considered an intermediate variable, the VA type could potentially influence prognosis. Therefore, we compared prognosis and hospitalization expenses between patients with or without a prepared VA and divided them into arteriovenous fistula or arteriovenous graft subgroups. As emergent hemodialysis (HD) initiation is associated with prognosis, we compared prognosis and hospitalization expenses between patients with or without a prepared VA according to emergent HD initiation.

**Online Resource 2. Causes of mortality among elderly patients (≥70 years)**

|  | All | Prepared VA (+) | Prepared VA (−) |
| --- | --- | --- | --- |
|  | (N = 22) | (n = 13) | (n = 9) |
| Cause of mortality |  |  |  |
| Cardiovascular disease (n,%) | 6 (27.3) | 5 (38.5) | 1 (11.1) |
| Infection (n,%) | 4 (18.2) | 3 (23.1) | 1 (11.1) |
| Malignant tumor (n,%) | 1 (4.5) | 1 (7.7) | 0 (0.0) |
| Others (n,%) | 3 (13.6) | 0 (0.0) | 3 (33.3) |
| Unknown (n,%) | 8 (36.4) | 4 (30.7) | 4 (44.4) |

VA: vascular access

**Online Resource 3. Causes of mortality among non-elderly patients (<70 years)**

|  | All | Prepared VA (+) | Prepared VA (−) |
| --- | --- | --- | --- |
|  | (N = 8) | (n = 3) | (n = 5) |
| Cause of mortality |  |  |  |
| Cardiovascular disease (n,%) | 3 (37.5) | 2 (66.7) | 1 (20.0) |
| Infection (n,%) | 0 (0.0) | 0 (0.0) | 0 (0.0) |
| Malignant tumor (n,%) | 2 (25.0) | 0 (0.0) | 2 (40.0) |
| Others (n,%) | 1 (12.5) | 1 (33.3) | 0 (0.0) |
| Unknown (n,%) | 2 (25.0) | 0 (0.0) | 2 (40.0) |

VA: vascular access

**Online Resource 4. Cox hazards regression analysis to determine the interaction between the presence of prepared VA and age with regard to all-cause mortality**

|  | Presence of prepared VA and age | | |
| --- | --- | --- | --- |
|  | HR | 95% CI | P-value for interaction |
| All patients | 1.09 | 1.01–1.18 | 0.021 |
| Elderly (≥70) | 1.11 | 0.94–1.32 | 0.22 |
| Non-elderly (<70) | 0.26 | 0.89–1.33 | 0.42 |

Statistical significance was set at P < 0.05.

CI: confidence interval, HR: hazard ratio, VA: vascular access

**Online Resource 5. Logistic regression analysis to determine the interaction between the presence of prepared VA and age with regard to high hospitalization expenses (>1,300,000)**

|  | Presence of prepared VA and age | | |
| --- | --- | --- | --- |
|  | HR | 95% CI | P-value for interaction |
| All patients | 1.06 | 0.97–1.16 | 0.15 |
| Elderly (≥70) | 1.13 | 0.89–1.43 | 0.33 |
| Non-elderly (<70) | 1.18 | 0.94–1.48 | 0.16 |

Statistical significance was set at P < 0.05.

CI: confidence interval, OR: odds ratio, VA: vascular access

**Online Resource 6. Comparison of survival rates between patients with and without a prepared AVF or AVG divided into non-elderly and elderly groups**

**
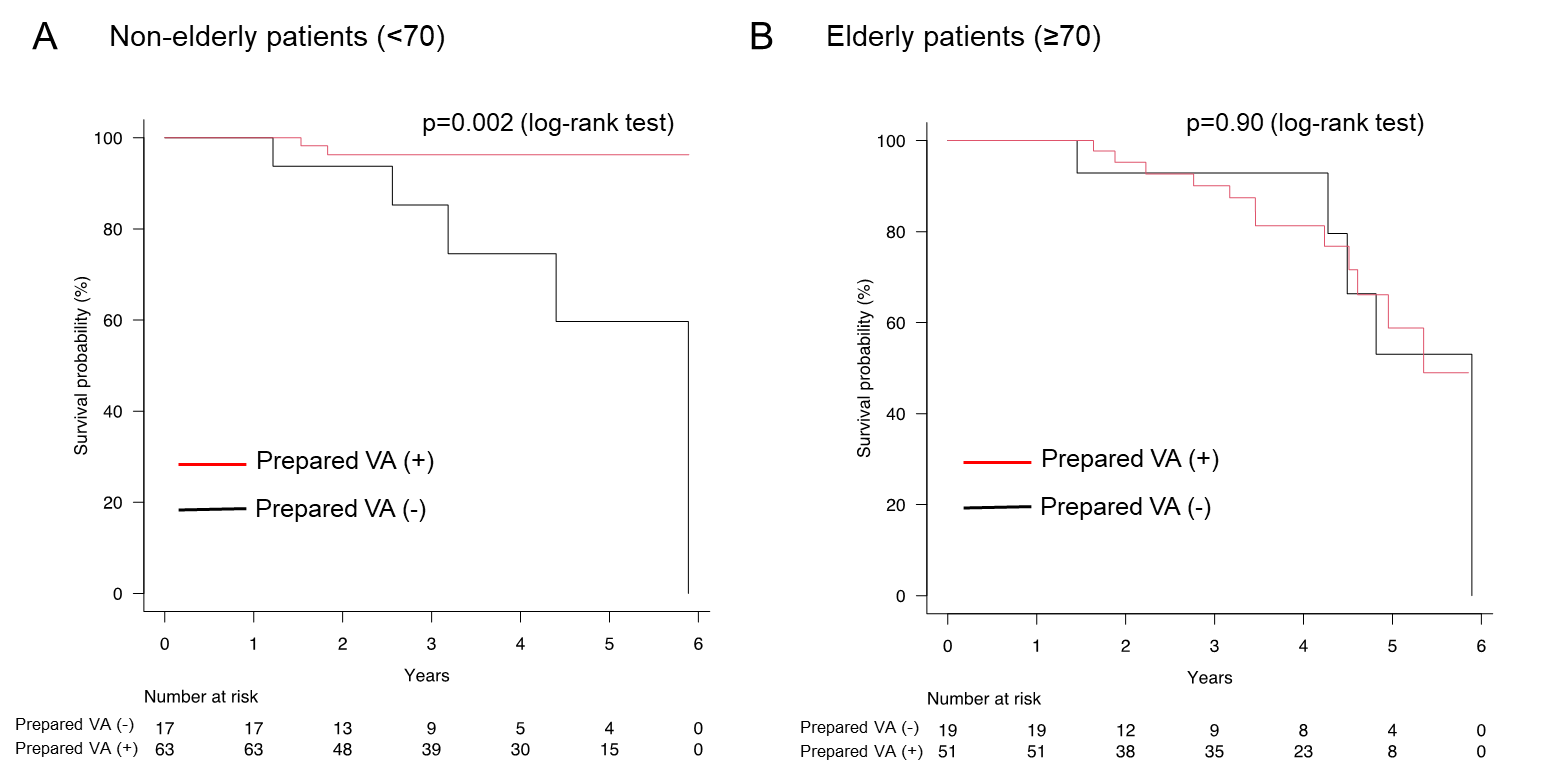
**

The survival rate was evaluated using the Kaplan–Meier method and log-rank test.

A: Comparison of survival rates among non-elderly patients with CKDG5 with and without a prepared AVF or AVG. B: Comparison of survival rates among elderly patients with CKDG5 with and without a prepared AVF or AVG. VA: vascular access

**Online Resource 7. Univariate logistic regression analyses of factors associated with high hospitalization expenses (>1,300,000) in patients whose final VA was an arteriovenous fistula (AVF) or arteriovenous graft (AVG)**

|  | Elderly (≥70) | | |  | Non-elderly (<70) | | |
| --- | --- | --- | --- | --- | --- | --- | --- |
|  | OR | 95% CI | P-value |  | OR | 95% CI | P-value |
| Presence of prepared VA (unadjusted) | 0.05 | 0.01–0.45 | 0.006 |  | 0.06 | 0.02–0.27 | <0.001 |

Statistical significance was set at P < 0.05.

CI: confidence interval, OR: odds ratio, VA: vascular access

**Online Resource 8. Comparison of survival rates among patients requiring emergent HD with and without a prepared VA and divided into the non-elderly and elderly groups**

**
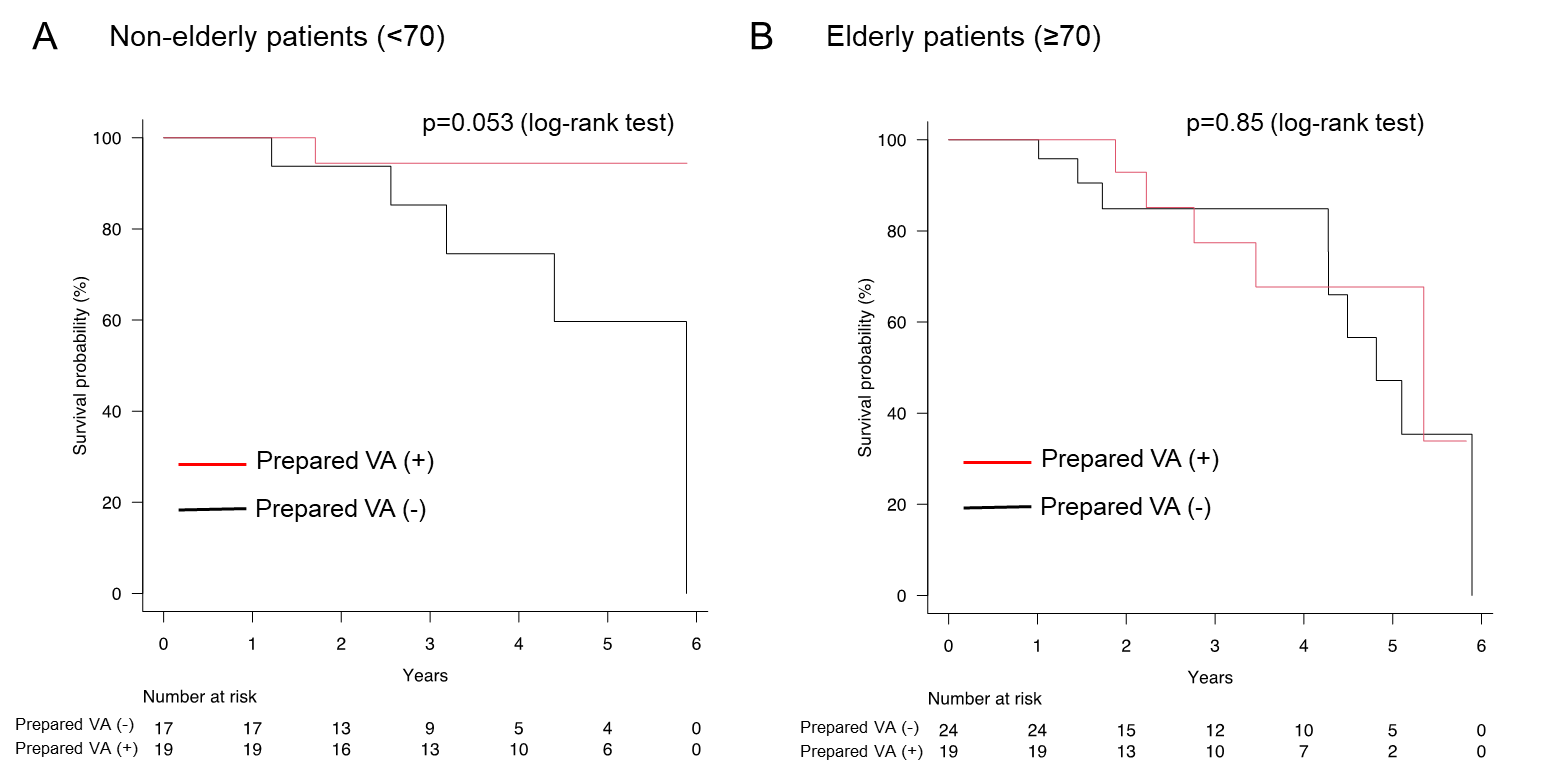
**

The survival rate was evaluated using the Kaplan–Meier method and log-rank test.

A: Comparison of survival rates between non-elderly patients with and without a prepared VA requiring emergent HD. B: Comparison of survival rates between elderly patients with and without a prepared VA requiring emergent HD. VA: vascular access

**Online Resource 9. Univariate logistic regression analyses of factors associated with high hospitalization expenses (>1,300,000) in patients who underwent emergency hemodialysis**

|  | Elderly (≥70) | | |  | Non-elderly (<70) | | |
| --- | --- | --- | --- | --- | --- | --- | --- |
|  | OR | 95% CI | P-value |  | OR | 95% CI | P-value |
| Presence of prepared VA (unadjusted) | 0.26 | 0.05–1.25 | 0.09 |  | 0.27 | 0.05–1.35 | 0.11 |

Statistical significance was set at P < 0.05.

CI: confidence interval, OR: odds ratio, VA: vascular access
